# Queen execution in a monogynous ant

**DOI:** 10.1002/ece3.7173

**Published:** 2021-01-20

**Authors:** Julia Giehr, Jürgen Heinze

**Affiliations:** ^1^ Zoology/Evolutionary Biology University of Regensburg Regensburg Germany

**Keywords:** altruism, ants, matricide, queen execution, *Temnothorax*, worker reproduction

## Abstract

Workers in many species of social insects are capable of laying unfertilized eggs, which can develop into haploid males. This causes a conflict about male parentage between queens and workers. In a few species, this may result in matricide, that is, workers kill the colony's queen. Queen killing has so far been observed mainly in multi‐queen colonies or in annual species, when the queen's fecundity declines at the end of the reproductive period. Here, we report queen expulsion and matricide in a monogynous, monandrous ant with perennial societies. Workers were seen to aggressively expel both related and unrelated queens from their nest shortly after the end of hibernation. Queen expulsion and matricide led to a significant decrease in the number of workers and brood, but eventually increased the direct fitness of workers through significant male production. Long‐term observations revealed a short lifespan of queens, while workers in orphaned colonies survived and produced male offspring over several years.

## Graphical abstract

Workers of the ant Temnothorax crassispinus have regularly been observed to attack and kill the only queen present in their colonies in spring. This explains the large fraction of queenless colonies in nature and might increase the share of workers in the males produced in the population.

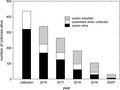

## 1 INTRODUCTION

The societies of social insects are often seen as harmonious, well‐organized units, in which all individuals cooperate smoothly to increase the reproductive output of the group as a whole. However, insect societies are not free of conflict. One prominent example is the conflict between queens and workers of social Hymenoptera (ants, bees, wasps) about the production of male offspring (Bourke, 1988a; Hammond & Keller, 2004; Heinze, 2004; Ratnieks, 1988; Ratnieks et al., 2006; Ratnieks & Reeve, 1992). Despite of anatomical constraints, such as the lack of a spermatheca for the storage of sperm and a reduced number of ovarioles, workers of many species are capable of increasing their direct fitness by rearing haploid sons from their unfertilized eggs (Bourke, 1988b; Choe, 1988; Helanterä & Sundström, 2007). Worker reproduction can be costly for the colony, as egg‐laying workers engage less in nonreproductive tasks (Bocher et al., 2008; Bourke, 1988a; Dampney et al., 2004; Tsuji et al., 2012), and queens and also workers often prevent other workers from oviposition by policing (Keller & Nonacs, 1993; Le Conte & Hefetz, 2008; Ratnieks, 1988; Ratnieks & Wenseleers, 2005; Stroeymeyt et al., 2007).

In a few cases, however, workers eliminate or attack their own queen and begin to lay eggs in its place. Such matricide has been observed in the annual societies of bumblebees and wasps when the queen's fecundity declines in late summer (Almond et al., 2019; Bourke, 1994; Loope, 2015, 2016; Strassmann et al., 2003). Queens of monogynous ants may live for several years (Keller, 1998; Keller & Genoud, 1997; Plateaux, 1971), and it is therefore not expected that workers kill the only individual in the nest that is capable of producing female offspring. Queen killing in societies of perennial social insects has so far been reported only from polygynous ants and stingless bees, where surplus young queens are eliminated (Balas, 2005; Inoue et al., 2015; Keller et al., 1989; Wenseleers et al., 2004), when new colonies of monogynous species are cooperatively initiated by several foundresses (Bernasconi & Strassmann, 1999; Forsyth, 1980; Heinze, 1993), or when matched‐mated queens produce large numbers of diploid males instead of workers (Vollet‐Neto et al., 2019). Furthermore, in the raider ant *Ooceraea biroi* workers, which produce female offspring via thelytokous parthenogenesis out of synchrony with other reproductives, may be attacked and killed by their nestmates (Teseo et al., 2013).

Here, we report on matricide in mature colonies of the monogynous, monandrous ant *Temnothorax crassispinus*. We examined worker reproductive success over several years in originally queenright colonies and colonies, in which workers had actively expelled and/or killed their queen (“orphaned colonies”). Queens of monogynous *Temnothorax* have been observed to have a mean survival time in the laboratory of 5.9–15 years (Keller, 1998; Plateaux, 1971), but previous studies indicated a high queen turn over and frequent colony usurpations and fusions in *T. nylanderi* (Foitzik & Heinze, 1998, 2000) and its sibling species *T. crassispinus* (Giehr et al., 2020; Giehr, Wallner, et al., 2020; Strätz & Heinze, 2004).

To determine queen lifespan under natural climatic conditions, we collected more than 400 colonies of *T. crassispinus* during hibernation in early spring 2016 and kept them in artificial nests outside the building over 5 years and throughout this time monitored queen survival. Our study shows that in a large number of colonies workers attacked and finally killed or expelled their queens at the beginning of the reproductive season in late spring. Similar to queenright colonies, about half of these orphaned colonies survived until the fourth year after collection and during this time produced large numbers of male offspring.

## 2 MATERIAL AND METHODS

### 2.1 Study species and collection of colonies

Colonies of *Temnothorax crassispinus* (Karavajev 1926) consist of only a few dozens or hundreds of workers and a single, singly mated queen. Colony structure is relatively fluid, as colonies may seasonally split and merge, adopt alien workers or are usurped by alien queens (Giehr, Wallner, et al., 2020). Nests are found in acorns and rotting twigs in deciduous forests throughout Eastern Central and Eastern Europe (Mitrus, 2015; Seifert, 2018; Strätz & Heinze, 2004; Tichá & Štys, 2002). In total, we collected 436 colonies around Regensburg, Germany. Data about male paternity and genetic structure of these colonies are analyzed in a separate manuscript, in which also details about collecting and colony maintenance are given (Giehr, Wallner, et al., 2020). In short, individual colonies were transferred from their natural nests into separate plastic boxes (10 × 10 × 3 cm^3^) with a plaster floor and a Plexiglas^®^ frame sandwiched between two microscope slides serving as nest. Colonies were kept under natural temperature and humidity conditions in a large rabbit hutch on the Western side of the university building, vis‐à‐vis a grassy slope and an open deciduous forest, in which *T. crassispinus* naturally occurs. Colonies were fed with pieces of cockroaches and honey twice per week in spring and summer, once per week at day temperatures below 15°C, and once every two weeks at day temperatures below 5°C. Nests were protected with fleece at temperatures below 0°C (see also Giehr, Wallner, et al., 2020).

Worker number and brood items were counted immediately after collection and again approximately 5, 10, and 20 weeks after colony collection during the first year (2016). Furthermore, the status of colonies was regularly checked during the first months after collection and again in spring 2020, and aggression against queens or the presence of injuries was noted. In 2017, 2018, and 2019, we noted queen survival, worker number, and the number of brood items once per year in summer, when the caste and sex of pupae could be identified. In these years, aggression toward queens was not studied in order to keep the colonies as undisturbed as possible. Female sexuals do not mate in laboratory nests but nevertheless may shed their wings. As we did not remove individuals from the colonies except for genetic analysis, colonies typically contained unmated, dealate female sexuals together with the dealate fertile queen.

### 2.2 Microsatellite analysis

To determine whether attacked queens might have been unrelated usurper queens, we investigated the microsatellite genotypes of 7–12 workers or, to keep workers for subsequent breeding periods, female sexuals and the queen from each of 9 and 13 colonies, which had expelled their queen in spring 2016 and 2017, respectively. Details on microsatellite analyses are given in Giehr, Wallner, et al. (2020). In short, we extracted DNA using the CTAB method (modified from Sambrook and Russell (2001) and amplified six DNA at six microsatellite loci by PCR (GT218, Hamaguchi et al., 1993; 2MS17, Suefuji et al., 2011; L5, Foitzik et al., 1997; L18, Foitzik et al., 1997; Ant3993, Butler et al., 2014, GT1 (Bourke et al., 1997). PCR products were analyzed in an ABI PRISM 310 Genetic Analyzer (PE Biosystems) after denaturation at 90°C for 1 min. Allele sizes were determined using GENESCAN 3.1 software (PE Biosystems).

The relationship between queens and the colony's workers was deduced by comparison of multilocus genotypes. Queens were defined as “alien” when their alleles did not match the presumed maternal and paternal alleles of the majority of the colony's workers at two or more loci (see also Giehr, Wallner, et al., 2020). Queen–worker relatedness was calculated using GenAlEx 6.5 (Peakall & Smouse, 2006, 2012), and standard errors were obtained by jackknifing over colonies.

Data were analyzed using R 3.2.3 software (R Development Core Team, 2008).

## 3 RESULTS

When collected in spring 2016, 320 of 436 colonies (73%) contained a queen, but almost half of the queens (48%, 152/320) died before the next hibernation in winter 2016/2017. A large fraction of these queens (78%, 119/152) left the nest because they were attacked or actively killed by workers 1–6 weeks after collection. In these colonies, one or several workers were observed to bite the legs of the queen or to cut off its limbs or head. Workers actively prevented expelled queens from re‐entering the nest by mandible threats and biting.

**FIGURE 1:**
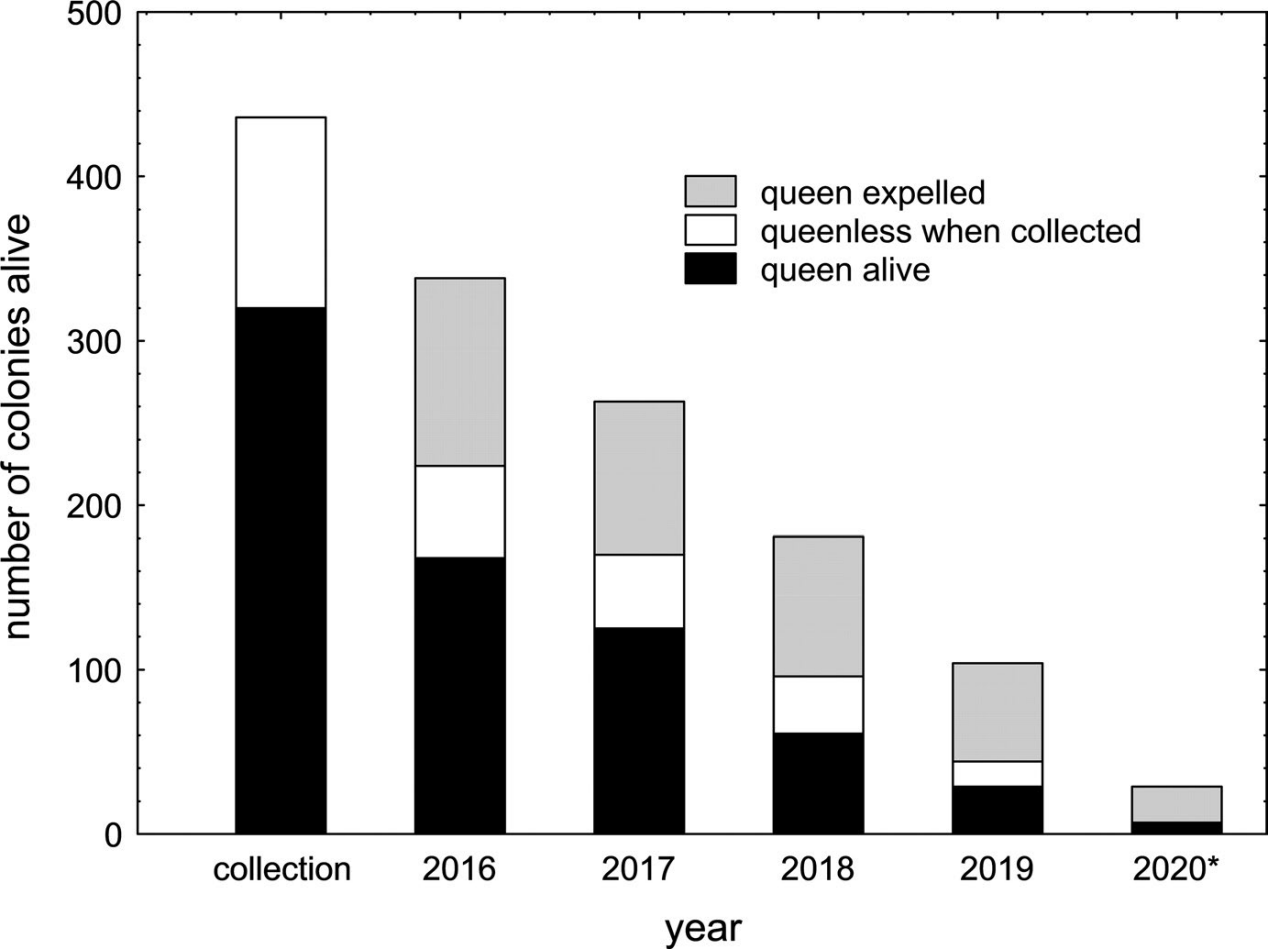
Changes in the number of live colonies of the ant *Temnothorax crassispinus* that were collected either queenless or without queen in spring 2016 and were kept under natural conditions in artificial nests. In a considerable number of colonies, workers expelled or killed queens, or queens died of other causes, leading to a decline in the number of queenright colonies. Similarly, the number of originally queenless colonies or colonies in which workers had expelled the queen decreased as workers died out with time

In 2017, 43 of the remaining 168 queens (26%) vanished or died, in 2018 64 of the remaining 125 queens (51%), and in 2019 32 of the remaining 61 queens (52%), that is, only 9% of the initially collected queens survived until summer 2019 (29/320). In summer 2020, only 7 colonies (2%) still contained a queen and produced female offspring (Figure 1). Whether queen death resulted from worker aggression was not investigated in 2017, 2018, and 2019, however, similar attacks against queens were observed in colonies collected in spring 2019 (L. Pedraza and A. Bernadou, pers. comm.). To confirm that queens are attacked also in colonies that had not been disturbed by recent collection and transfer into a new nest, observations were resumed in 22 colonies whose queens had survived until April 2020. In two colonies, we found a single, recently dead queen with cutoff tarsae. Dissection showed that both had a full spermatheca, eggs in development and corpora lutea, that is, they had been the mated egg layers of the respective colonies. In a third colony, two dealate female sexuals were seen fighting and biting off each other's antennae. Dissection revealed that one of them had been the mated egg layer, that is, queen, of the colony, while the spermatheca of the other individual was not visible. It presumably was an unmated female sexual, as sexuals of *T. crassispinus* do not mate in the laboratory. Queens survived in captivity for a mean of only 1 year (*n* = 320).

The genotypes of 11 of 27 attacked and genetically analyzed queens (14 in 2016, 13 in 2017; 44%, 22 colonies, four colonies contained two or three dealate female sexuals when collected in 2016), suggested that they were the mother of the majority of workers. Four queens, including one in a colony with two dealate female sexuals, might have been a sister of the present workers, the remaining 12 queens, including one, two, and three female sexuals in three colonies, appeared to be not related to the colony's workers and/or female sexuals. The proportion of queens that were not mothers of the majority of females in the nest did not differ between attacked queens and queens from natural colonies collected in summer 2016 (Giehr, Wallner, et al., 2020; 16 of 27 vs. 8 of 30; two‐sided Fisher's exact test, *p* = .103). The mean relatedness between the attacked queens and their nestmate workers or winged female sexuals was 0.226 ± *SE* 0.104 in 2016 (*n* = 9) and 0.417 ± 0.041 in 2017 (*n* = 13). The significantly higher mean in 2017 (two‐tailed *t* test, *t* = 2.615, *p* = .017) presumably reflects the fact that experimental colonies could not be usurped by new unrelated queens and many more workers were now offspring of the present queen. Queens that were attacked and expelled in 2016 did not differ in their relatedness to workers from queens that were not attacked (mean 0.351 ± 0.065, *n* = 15, Giehr, Wallner, et al., 2020; *t* = 1.536, *p* = .139).

Colonies that had expelled their queens were significantly larger than other queenright colonies (Mann–Whitney *U* test, *U* = 9,448, *p* = .002, for details see Table 1). After expulsion and killing of the queen, worker number (*n* = 119 colonies, *U* = 13,177, *p* = .024) and brood number (*U* = 10,236, *p* = .001) decreased significantly over the next 4 weeks compared to still queenright colonies. Colonies, which had expelled their queen, initially contained more larvae (*U* = 7,237, *p* = .011) and eventually produced more eggs (*U* = 8,497, *p* < .0001) in summer 2016. This resulted in a higher number of larvae in fall 2016 (*U* = 7,956, *p* = .0002) than in queenright colonies. About half (53%, 107/201) of the initially queenright colonies, 50% (60/119) of the colonies that had expelled their queens in 2016, and 13% (15/116) of the originally queenless colonies still contained workers and survived until summer 2019.

**TABLE 1:** Summary of colony data (median, Q1, Q3) collected over a 4‐year period in queenright *Temnothorax crassispinus* colonies that remained queenright throughout the years and in colonies that had expelled their queen in 2016

	Queenright				Queen expelled in 2016			
	*n*	Median	Q1	Q3	*n*	Median	Q1	Q3
Worker number at collection	**200**	**150**	**100**	**213**	**119**	**180**	**128**	**230**
Proportion of workers left 4 weeks after queen expulsion	**197**	**0.73**	**0.60**	**0.96**	**116**	**0.69**	**0.53**	**0.86**
Worker number summer 2017	188	47	27	72	113	50	28	81
Worker number summer 2018	61	15	6	28	85	12	7	23
Worker number summer 2019	**29**	**16**	**9.5**	**28**	**69**	**6**	**2**	**9**
Number of larvae at collection	**172**	**122**	**80**	**220**	**103**	**180**	**100**	**270**
Proportion of larvae still present 4 weeks after queen expulsion	**166**	**0.91**	**0.60**	**1.40**	**100**	**0.69**	**0.47**	**1.02**
Eggs produced summer 2016	**197**	**53**	**12**	**120**	**117**	**100**	**40**	**240**
Larvae produced summer 2016	**187**	**250**	**136**	**395**	**114**	**368**	**198**	**615**
Larvae produced summer 2017	156	73	30	160	94	72	38	178
Males produced in summer 2016	195	1	0	5	113	1	0	6.5
Proportion of males in all sexual summer 2016	135	0.54	0.08	0.96	76	0.50	0.15	0.88
Males produced in summer 2017	30	47	17	91	28	94	70	150
Proportion of males in all sexual summer 2017	**30**	**0.83**	**0.55**	**0.94**	**28**	**0.93**	**0.85**	**0.96**
Males produced in summer 2018	58	26.5	6	49.25	85	19	6.5	54.5
Proportion of males in all sexual summer 2018	**58**	**0.78**	**0.62**	**0.91**	**73**	**1.00**	**1.00**	**1.00**
Males produced in summer 2019	29	27	4.5	48.5	69	24	8	52
Proportion of males in all sexual summer 2019	**29**	**0.68**	**0.19**	**0.92**	**63**	**1.00**	**1.00**	**1.00**

Note

Significant differences (Mann–Whitney *U* test, *p* < .05) between both groups were marked in bold (for details see results). In 2017, only a subset of the colonies was investigated.

The proportion of males in all sexuals varied significantly across all collected colonies (median, quartiles: queenright, *n* = 135, 0.538, 0.071–0.965; queen expelled, *n* = 76, 0.500, 0.143–0.882; originally queenless, *n* = 44, 0.813, 0.468–1.000; Kruskal–Wallis test, *H* = 7.005, *p* = .027; sequential Bonferroni significance of pairwise comparisons by Dunn's post hoc test, queenright vs. queenless, *p* = .016; queenright vs. queen expelled, *p* = .692; queen expelled vs. queenless, *p* = .012). The death or killing of the queen had no effect on the proportion of males among all sexuals produced in the same year, because sexual larvae of *Temnothorax* typically need to hibernate at least once before adult emergence (Buschinger, 1973).

**FIGURE 2:**
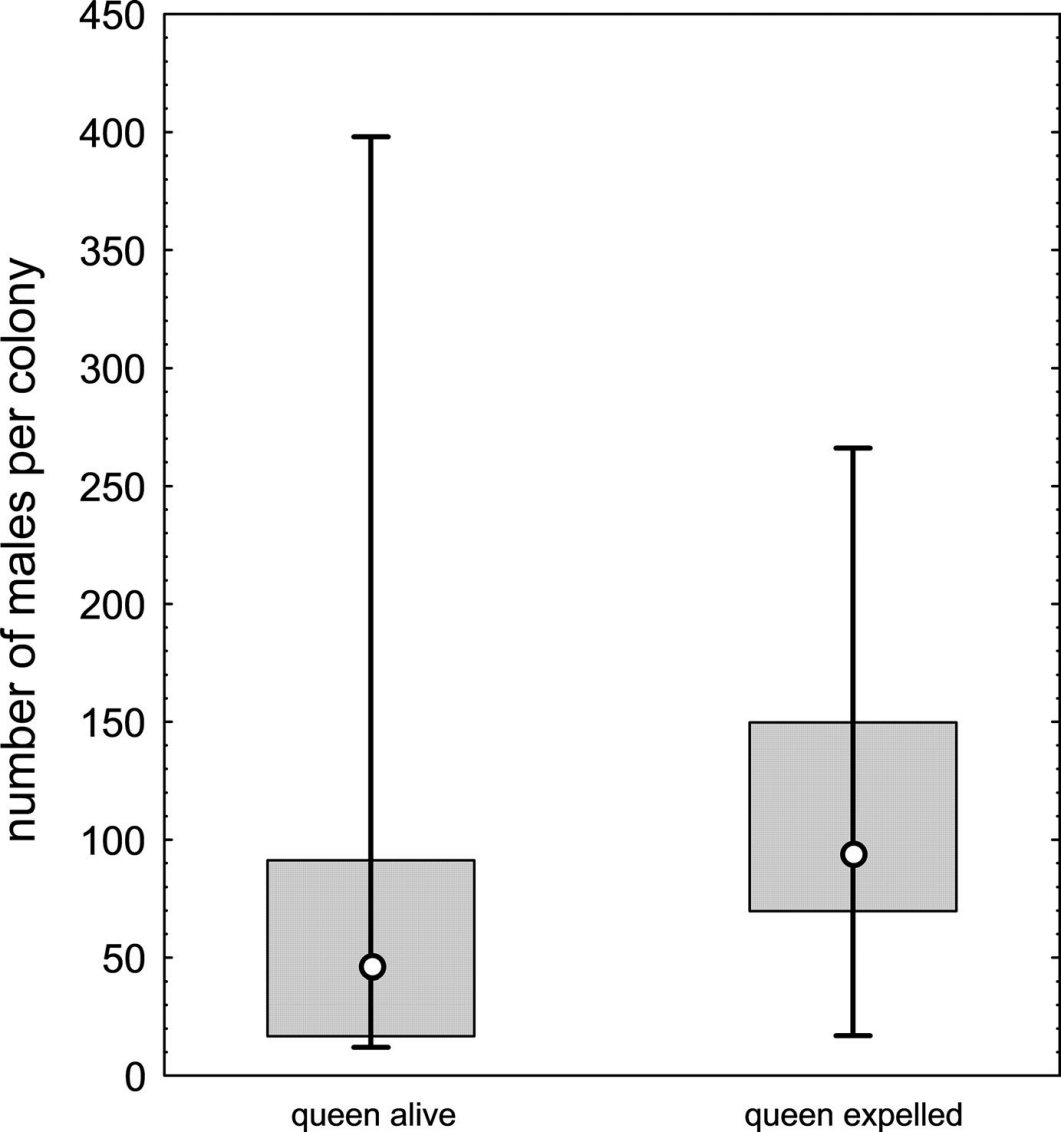
Change in the number of males produced in colonies of the ant *Temnothorax crassispinus* 1 year after workers had expelled or killed the present queen (median, quartiles, range)

In the year following collection (2017), the number of workers (*U* = 9,998, *p* = .393) and the number of larvae (*U* = 6,758, *p* = .300) did not differ between queenright colonies and colonies that had expelled their queen, but queenless colonies produced significantly more males than queenright colonies (Figure 2, *U* = 267, *p* = .008) and a significantly more male‐biased sex ratio (*U* = 257, *p* = .011). As expected, queenless colonies produced only male offspring in 2018 and 2019.

Though we provided colonies with food ad libitum and kept them under natural climatic conditions, worker numbers declined over time in both queenright and queenless colonies (Wilcoxon signed rank tests: 2016 vs. 2017: *V* = 60,601, *p* < .0001; 2017 vs. 2018: *V* = 31,058, *p* < .0001; 2018 vs. 2019: *V* = 30,202, *p* < .0001).

## 4 DISCUSSION

Queen–worker conflict about the origin of males in insect societies is typically resolved by queen pheromones and / or aggression toward egg‐laying workers by the queen and other workers (policing; Hammond & Keller, 2004; Heinze, 2004; Ratnieks et al., 2006). In the ant *Temnothorax crassispinus*, genetic data suggest a considerable contribution of workers to the males in both queenless and queenright colonies (Giehr, Wallner, et al., 2020). Here we show that worker reproduction may additionally benefit (a) from a relatively short lifespan of queens and (b) worker aggression toward queens. Both lead to at least temporary queenlessness of colonies and though workers have been shown to be also capable of rearing sons in the presence of the queen (El‐Shehaby et al., 2012; Giehr, Wallner, et al., 2020), a boost of worker egg laying. Furthermore, frequent queenlessness might also promote the usurpation of colonies by alien queens and colony fusion, explaining the genetic heterogeneity of natural colonies (Giehr, Wallner, et al., 2020).

In our experiment, only 29 of 320 of the collected queens survived for more than 3 years after collection under natural climatic condition. This differs considerably from previously reported mean lifespans of related species in the laboratory, which range from 5.9 to 15 years (Keller, 1998). Though we only observed colonies for worker aggression in the first and last year of the study, it is likely that queen killing and expulsion contributed to the high mortality also in the other years. Workers began to expel and kill the queens one to six weeks after colony collection in early spring. Contrary to previous observations in polygynous Argentine ants, in which queen executions apparently serve to increase the relatedness among workers (Inoue et al., 2015), aggression in *T*. *crassispinus* was not preferentially directed toward unrelated queens, which had usurped a colony and replaced the resident queen. Furthermore, as queen killing occurred in early spring, when queens began to reactivate their ovaries, it is unlikely that aggression was triggered by decreasing queen fecundity, as observed in wasps and bumblebees (Bourke, 1994; Loope, 2015, 2016). Similarly, there is no evidence that queen removal was based on poor health of the queen: In a previous study, we did not observe any aggression against diseased or dying queens. Instead, such queens were intensively groomed by workers even after their deaths (Giehr & Heinze, 2018).

At present, we cannot completely exclude that queen executions in the first year were a delayed consequence of colony collection, for example, workers attacking queens, which had been injured during collection or which began to lay eggs and thus changed their cuticular odor bouquet in the still unfamiliar, artificial environment. Several observations speak against this hypothesis: First aggression occurred only several weeks after colonies had moved into the artificial nests; attacks against queens were never observed in colonies collected in summer (*n* > 80 colonies, J. Giehr, unpubl.); and queens died at a similar rate during the following springs even though their nests had not been disturbed. Finally, similar aggression as in the first year appeared to occur also in the last year of our study period. According to a suggestion by B. Seifert (pers. comm.), global warming might have negatively affected the condition of queens, to which workers may have reacted by aggression. Indeed, unusually hot and dry summers and warm winters have been shown to decrease the productivity of ant queens (e.g., Seifert, 2018), and in annual bumblebees, weakening queens have been observed to be attacked by their workers (e.g., Almond et al., 2019; Bourke, 1994). However, as mentioned above, in an earlier study (Giehr & Heinze, 2018) we did not observe aggression against moribund, fungus‐infected queens even though they stopped laying eggs.

Future research will show whether queen execution is a consequence of global change or an adaptive strategy of *T. crassispinus* workers. In any case, queen death led to a large increase of worker reproduction. Although over the years, worker numbers decreased in orphaned colonies, workers produced numerous males during the following years and thus gained considerable fitness. Even though up to 20% of the males produced in queenright colonies may be worker offspring (Giehr, Wallner, et al., 2020), killing the queen might be an important additional strategy for workers to increase their direct fitness. The resulting male‐bias might be compensated by an increased production of female sexuals in colonies that contain or only recently lost a fertile queen. Indeed, sex ratios in queenright field colonies were highly split (see also Strätz & Heinze, 2004), with some colonies investing heavily in female sexuals.

## CONFLICT OF INTEREST

None declared.

## AUTHOR CONTRIBUTIONS

**Julia Giehr:** Conceptualization (equal); formal analysis (lead); funding acquisition (supporting); investigation (lead); writing–original draft (lead); writing–review and editing (supporting). **Jürgen Heinze:** Conceptualization (equal); formal analysis (supporting); funding acquisition (lead); investigation (supporting); writing–original draft (supporting); writing–review and editing (lead).

## ETHICAL APPROVAL

*Temnothorax crassispinus* is an unprotected ant species. All experiments comply with European laws.

## Data Availability

Raw data, individual genotypes, and detailed pairwise relatedness analysis are available at https://doi.org/10.5061/dryad.f4qrfj6v6
